# Fully automated detection and identification of CSF shunt valves using YOLOv8 and a class-based reference image assignment as a safety mechanism

**DOI:** 10.1038/s41598-025-29201-0

**Published:** 2025-11-21

**Authors:** Mathias Holtkamp, Jannis Straus, Luca Salhöfer, Hanna Styczen, Maharani Budi Santoso, Sebastian Zensen, Cornelius Deuschl, René Hosch, Michael Forsting, Yan Li, Lale Umutlu, Felix Nensa, Johannes Haubold

**Affiliations:** 1 Institute of Diagnostic and Interventional Radiology and Neuroradiology, University Hospital Essen, Essen, Germany; 2 Institute for Artificial Intelligence in Medicine (IKIM), University Hospital Essen, Essen, Germany

**Keywords:** Hydrocephalus, Cerebrospinal fluid shunts, Radiography, Deep learning, Image processing, Computer-assisted, Hydrocephalus, Neurological disorders

## Abstract

The study aimed to develop and evaluate an algorithm based on the YOLOv8x framework to automatically detect and identify cerebrospinal fluid (CSF) shunt valves. This approach seeks to streamline the diagnostic process identifying shunt valve types and pressure levels. A retrospective cohort of 2701 anonymized radiographs comprising six types of CSF shunt valves was used. Data augmentation techniques such as flipping, scaling, and mosaic augmentation were applied during training to enhance robustness. The dataset was split into 80% training and 20% testing subsets as part of a 5-fold cross-validation. Validation was conducted on a separate test set of 295 images using metrics such as mean Average Precision (mAP) at intersection over union thresholds of 50% (mAP50) as well as precision, recall, and F1-scores as metrics. Additionally, a class-based reference image assignment system was used to link the detected valves with the corresponding manufacturer images. These paired images were then independently reviewed by two radiologists to assess the accuracy of the algorithm’s classifications. The algorithm achieved a weighted mAP50 of 0.884 and a weighted average F1-score of 94.8%. High F1-scores were observed for Codman Certas (99.6%) and Codman Hakim (99.6%), with lower scores for less common valves like proGAV (30.8%). Radiologists were able to identify both correct and incorrect classifications made by the algorithm with 100% accuracy, due to the integrated safety mechanism. This safety mechanism relies on the fully automated linking of detected valves with the corresponding manufacturer images. In Conclusion the automated system demonstrated high efficiency in detecting and classifying CSF shunt valves, significantly simplifying the diagnostic workflow. Moreover, the integration of a robust safety mechanism ensures that potential misclassifications are identified and corrected.

**Supplementary Information:**

The online version contains supplementary material available at 10.1038/s41598-025-29201-0.

## Introduction

Hydrocephalus is a condition with a wide range of potential causes, which can be categorized into congenital and acquired origins^1^. Pathophysiologically, three mechanisms are identified as contributors to the development of hydrocephalus: obstruction of cerebrospinal fluid (CSF) flow, overproduction of CSF, or impaired CSF resorption^1–3^. The disturbance of the CSF leads to a dilation of the brain ventricles, which can lead to visual disturbance, ataxia, hemiparesis, epilepsy, headache and even cerebral edema^1,2,4–6^. The clinical condition has many possible causes and often affects children^2^. A common method of treating hydrocephalus is a CSF shunt. Using CSF shunt, excess CSF from the cerebral internal CSF spaces is drained into the peritoneal cavity or the vascular system^1,4^. Today, CSF shunt valves often consist of three parts: An inflow catheter, a valve mechanism, and an outflow catheter. The inflow catheter is positioned with its tip in the internal CSF spaces. CSF is directed through the inflow catheter to the shunt valve, which regulates pressure and controls outflow. The shunt valve is followed by the drain catheter, which directs the CSF into the target region. There are over 125 different shunt valves currently available, some of which are based on different mechanisms^7^. All shunt valves rely on a pressure difference to enable CSF drainage. Some valves, however, have a fixed opening pressure, while others are adjustable after installation, allowing for external modification of the pressure settings, for example, using a magnet^7^.

Previous studies have explored deep learning approaches for identifying CSF shunt valves in radiographs. Giancardo et al. achieved high classification accuracy but required manual selection of the valve region^8^. Sujit et al. used mobile phone photos of workstation screens, though their model was limited to three valve types^9^. Other approaches employed convolutional neural networks (CNN) for valve detection on skull X-rays but typically lacked integration with verification mechanisms such as visual matching with manufacturer reference images^10,11^. Fully automated systems with integrated visual verification mechanisms are not known in the current literature.

The setting of such a valve can change, for example, due to MRI examinations. An unintended change in the pressure level may alter the intracranial pressure. Pressure level of subcutaneously implanted shunt valves is routinely assessed by a cranial X-ray after every MR-examination.

Due to the number of different CSF valve types, reading the correct pressure levels often requires time-consuming literature research by the radiologist, as shunt valves vary in appearance and require different methods for determining the pressure levels, with no standardized visualization available.

Unlike previous approaches, this study aimed to develop the first fully automated pipeline that not only detects and classifies CSF shunt valves in radiographs using deep learning, but also integrates a class-based reference image assignment system (CBRIA) to enable visual verification and streamline pressure level interpretation.

## Methods

### Study design and cohort

A retrospective single-center study was performed using two datasets of partial skull X-ray images identified through a keyword search for “VP shunt” in radiological reports. All radiographs were acquired at the investigating hospital.

The first dataset consisted of 2701 radiographs collected between 01.01.2011 and 31.12.2019. These images were used for training and testing the model through a 5-fold cross-validation with an 80:20 split between training and testing in each fold. The dataset included the following six classes of CSF shunt valves: Codman Certas (974), Codman Hakim (1157), Codman Uni-Shunt (451), paediGAV (91), proGAV (13), and proGAV with gravitational unit (15).

**Fig. 1:**
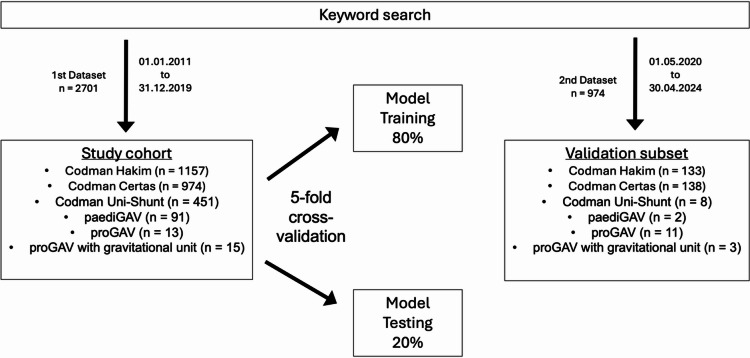
Overview of the study cohort composition. Radiographs of CSF shunt valves were identified through a keyword search and included in the study cohort (*n* = 2701). The cohort was divided into 80% for model training and 20% for model testing. Additionally, a validation subset (*n* = 295) was created.

The second dataset included 974 radiographs collected between 01.05.2020 and 30.04.2024, identified through the same keyword search. A subset of 295 radiographs was extracted from this dataset for model validation, maintaining a similar distribution as the training and test datasets. The validation subset comprised six shunt valve types: Codman Certas (138), Codman Hakim (133), Codman Uni-Shunt (8), paediGAV (2), proGAV (11), and proGAV with gravitational unit (3) (Fig. 1).

Although both datasets originate from the same clinical setting, they were collected in temporally distinct periods and handled separately to avoid temporal bias. The first dataset was exclusively used for model training and internal evaluation, while the second dataset served as an independent validation cohort to assess the generalizability of the trained model to unseen data.

### Data annotation

The annotation of CSF shunt valves in the X-ray images was performed using the open-source tool LabelImg^12^. Bounding boxes were manually drawn around the shunt valves in each image, creating precise annotations to serve as ground truth for training and evaluating the object detection model. Each valve was assigned to its class.

### Network

**Fig. 2:**
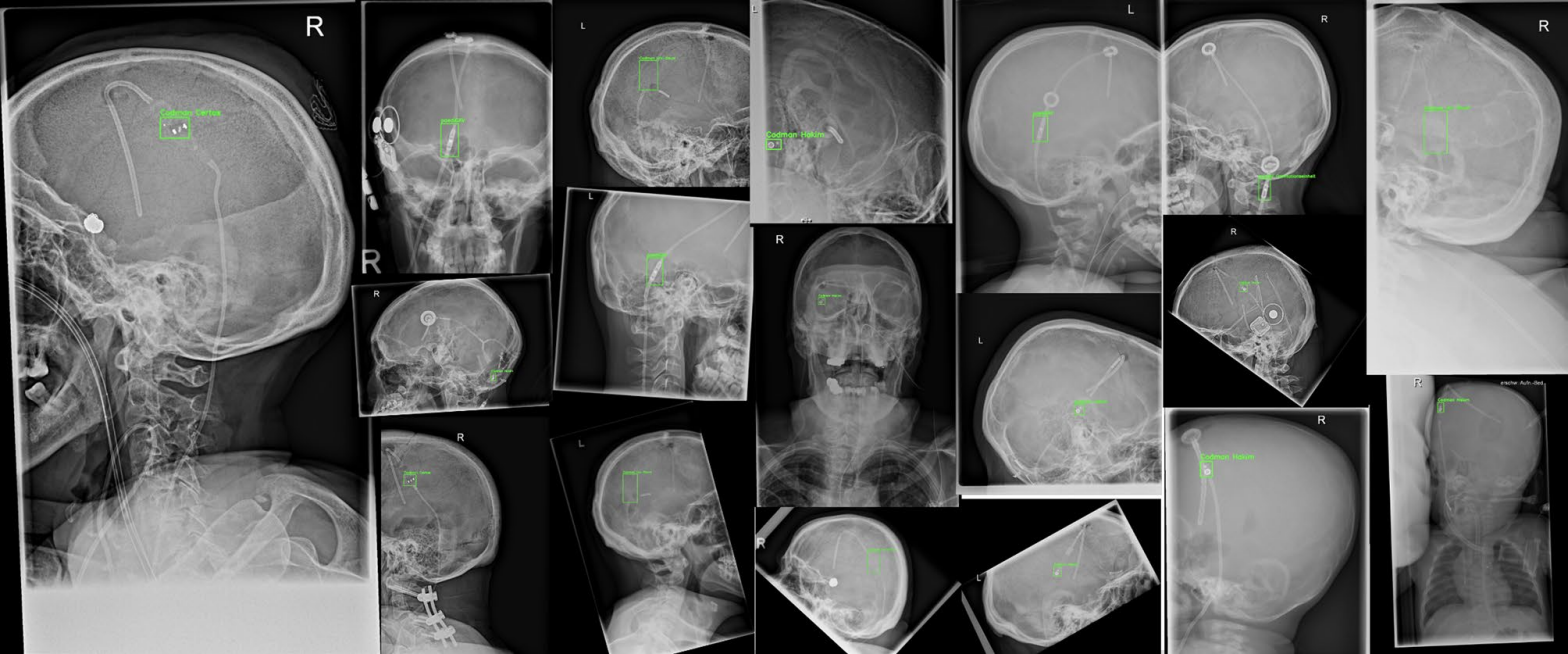
Representative X-ray images from the dataset, each showing a detected CSF shunt valve marked with the predicted bounding box.

A You Only Look Once (YOLO)v8x model was used as the network for object detection and classification of CSF shunt valves in X-ray images. YOLO is an advanced single-stage object detection model that employs a CNN backbone to extract feature maps from input images^13^. It predicts bounding boxes and class probabilities directly from these feature maps in a single pass, enabling real-time object detection^13^. Bounding boxes serve as a visible frame for object detection (Fig. 2) and show radiologists the region of interest (ROI) used by the network to classify the valve. YOLOv8x simultaneously performs object detection and classification, allowing for efficient identification and localization of shunt valves in X-ray images. The network was initialized with publicly available YOLOv8x weights pre-trained on the COCO (Common Objects in Context) dataset, which contains a large number of natural images with diverse object classes.

### Data augmentation

To improve the robustness and generalization of the detection model, standard augmentation techniques provided by the YOLOv8x framework were applied during training. These included horizontal flipping, scaling, translation, color augmentation, mosaic augmentation, and random erasing. All augmentations were applied dynamically and uniformly across the training data and were not intended to address class imbalance.

### Training protocol

Each fold was trained for 100 epochs with a batch size of 16 using the Ultralytics-YOLOv8 default optimiser (SGD with cosine learning-rate schedule, mixed precision). Loss-term weights were box 7.5, cls 0.5, dfl 1.5. Training was performed on a single NVIDIA RTX A6000 GPU and finished in ~ 50 min per fold. All network and training hyperparameters were defined prior to cross-validation and kept identical across all folds; no hyperparameter tuning was performed during the cross-validation process.

### Validation

The task of identifying CSF shunt valves in X-ray images is both a localization and an identification problem. Localization involves determining the exact position of the shunt valve within the image, while identification entails correctly assigning the detected valve to one of the predefined categories. To evaluate the accuracy of the developed method for the identification of CSF shunt valves, a 5-fold cross-validation was performed. The dataset was divided into five equal subsets, with one subset serving as the test set and the remaining subsets serving as training data in each iteration. For final predictions on the validation cohort, a majority voting approach was employed: for each fold, the model’s prediction with the highest confidence was recorded, and the final classification for each image was determined by selecting the label that appeared most frequently among the five folds.

The mean average precision (mAP) value at an intersection over union (IoU) of 0.5 was utilized as the primary metric for predicting localization accuracy. The IoU indicates the amount of overlap between a prediction box and a ground truth box. When there is a total overlap the value is 1. The closer the value gets to 0, the less overlap there is^14^. The mAP is a commonly used standard metric for assessing the accuracy of an object detection model^15–17^. It is calculated by averaging the area under the precision–recall curve (Average Precision, AP) across all classes: mAP = (1 / N) × ∑ AP_i_, where N is the number of classes and AP_i_ is the AP for class i. Each AP_i_ summarizes the precision and recall performance of the model for that class at different confidence thresholds. In this study, mAP was calculated at an IoU threshold of 0.5, meaning that a prediction was considered correct if it overlapped with the ground truth by at least 50%.

Additionally, precision, recall, and F1-score were calculated to evaluate the model’s ability to correctly identify shunt valves.

This cross-validation approach was intended to ensure robust internal performance estimates using all available training data. The separate validation dataset was used solely as an independent benchmark to assess the model’s generalizability on unseen data.

### CBRIA and radiological evaluation

In our framework, each detected shunt valve was automatically linked to a reference image representing its predicted class. This process, which we refer to as CBRIA, does not involve feature-based similarity matching but instead relies on a rule-based mapping from predicted class to a predefined reference image. After applying the YOLOv8 model, the bounding box with the highest confidence score was selected in each fold, and the corresponding predicted class label was recorded. For each image, a majority voting approach was then applied across all five folds to determine the final class label. This label was directly mapped to a reference image with the same filename. The assignment process did not involve any similarity metrics or feature-based comparisons. The reference images were obtained directly from the official websites of the respective valve manufacturers. These images are considered the officially provided standard representations of each valve model and display the configuration and adjustment features as defined by the manufacturer. No additional quantitative validation was performed, as the images inherently represent the manufacturer-defined reference standard.

**Fig. 3:**
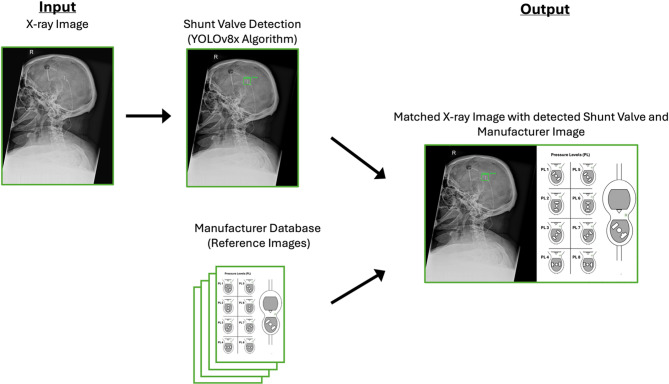
CBRIA for CSF Shunt Valve Detection starts with the input X-ray image, followed by shunt valve detection using the YOLOv8x algorithm. The detected valve is then assigned a corresponding manufacturer image based on its predicted class. The output combines the detected valve and the manufacturer’s image, which is schematically illustrated here as an example. This process enables radiologists to verify classifications and determine pressure levels efficiently.

This assignment mechanism serves two purposes: First, it enables fast and reliable identification of the pressure setting by displaying the exact valve configuration associated with the predicted class. Second, it provides a visual plausibility check, allowing radiologists to compare the manufacturer image with the detected valve. This contributes to transparency and supports the verification of automated predictions in line with current goals of explainable artificial intelligence in clinical imaging (Fig. 3).

For the validation cohort, consisting of 295 images, two radiology residents with 3 and 4 years of experience independently reviewed the paired images. For each X-ray image, they systematically assessed whether the shunt valve visible in the image matched the corresponding manufacturer image or whether a misclassification by the algorithm or an incorrect pairing by the CBRIA process had occurred.

### Preparation of the manuscript

For linguistic assistance in composing the manuscript, ChatGPT (Version GPT-4o), developed by OpenAI, was only used to improve the readability and language of the work.

## Results

In the evaluation of the model’s performance, both localization and identification of different CSF shunt valve types were assessed. The primary focus was on the accurate identification of the shunt valves, with localization considered a necessary step to support precise identification. The model identified the Codman Certas and Codman Hakim valves among six shunt valve types, with AP_50_ values of 0.919 and 0.947, respectively. For the remaining shunt valves, the mAP values ranged from 0.028 to 0.483 (Table 1). Overall, a weighted mAP_50_ of 0.884 were observed.

**Table 1:** Average precision (AP) at IoU thresholds of 50% (AP_50_) for each shunt valve model.

Class	*n*	AP_50_
Codman Certas	138	0.919
Codman Hakim	133	0.947
Codman Uni-Shunt	8	0.252
paediGAV	2	0.268
proGAV	11	0.483
proGAV gravitational unit	3	0.028

**Fig. 4:**
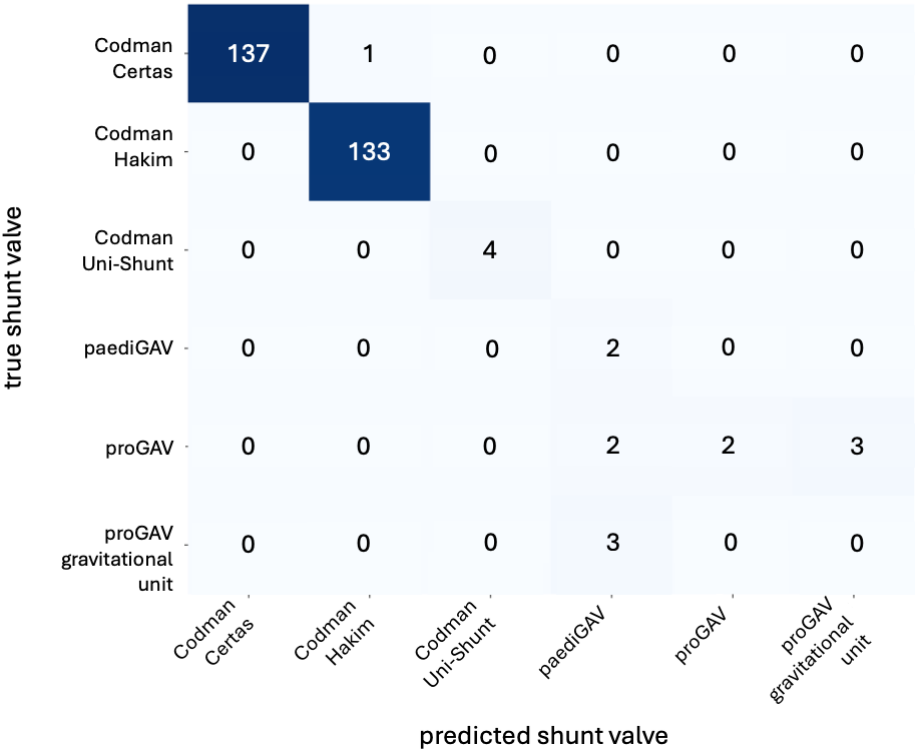
Confusion matrix illustrating the comparison between true and predicted shunt valve models within the validation dataset.

The model achieved a weighted average F1-score of 94.8% and a weighted average precision of 98.2% on 295 validation images. During validation, the algorithm correctly identified 278 out of 295 shunt valves, misclassified 9 valves, and failed to detect 8 valves. Examining the individual shunt valves revealed notable differences (Table 2; Fig. 4). High F1-scores were observed for the shunt valve types Codman Certas (99.6%) and Codman Hakim (99.6%), whereas the F1-score was reduced for the less common shunt valves: Codman Uni-Shunt (66.7%), paediGAV (44.4%), proGAV (30.8%), and proGAV gravity unit (0%).

**Table 2:** Performance metrics (Precision, Recall, and F1-score) for each shunt valve model.

Class	*n*	Precision	Recall	F1-score
Codman Certas	138	1.000	0.993	0.996
Codman Hakim	133	0.993	1.000	0.996
Codman Uni-Shunt	8	1.000	0.500	0.667
paediGAV	2	0.286	1.000	0.444
proGAV	11	1.000	0.182	0.308
proGAV gravitational unit	3	0.000	0.000	0.000

Two radiology residents with 3 and 4 years of experience, respectively, independently reviewed the automatically matched manufacturer’s images. All 295 images from the separate validation dataset, representing all six shunt valve types, were evaluated. Each image displayed the detected shunt valve, marked with a bounding box, alongside the corresponding reference image provided by the manufacturer. The radiologists assessed whether the algorithm’s classification matched the actual shunt valve type based on the displayed images. This process allowed them to evaluate the algorithm’s performance by identifying potential misclassifications or incorrectly matched reference images. Importantly, the radiologists were able to distinguish between correct and incorrect classifications in 100% of cases (Table 3). As a result, there were no instances where a misclassification by the algorithm went undetected, as the radiologists could reliably identify errors through direct comparison with the manufacturer’s images.

**Table 3:** Radiologists’ assessment of algorithm outcomes by classification result.

Outcome	Number of cases	Correctly identified	Reviewer accuracy (%)
Correctly classified valves	278	278	100
Misclassified valves	9	9	100
Missed detections	8	8	100
Total	295	295	100

The table shows the number of correctly classified, misclassified, and undetected (missed) shunt valves in the validation dataset (*n* = 295), along with the number of cases in which radiologists correctly identified each outcome. The “Reviewer Accuracy” column indicates the proportion of cases where the radiologists’ assessment correctly reflected the algorithm’s classification result.

## Discussion

In this study, our primary objective was to develop and evaluate an algorithm utilizing the YOLOv8x framework to automatically detect and classify CSF shunt valves on X-ray images and match them with corresponding manufacturer images. This approach aimed to optimize the diagnostic process of reading pressure values. The automatic matching with manufacturer images not only facilitates immediate reading of the pressure levels but also serves as an additional safety mechanism. This is because the direct comparison with the reference image allows misclassified shunt valves to be immediately identified.

In developing our framework, we deliberately chose an object detection model rather than an image-level classifier, as shunt valves typically occupy only a small portion of the radiograph and are embedded within a large amount of irrelevant anatomical background. Object detection focuses the model’s attention on the most relevant regions, which helps to reduce the influence of spurious correlations and irrelevant image content. Additionally, the bounding box provides a transparent visual cue that allows radiologists to assess whether the classification result is anatomically plausible. This supports interpretability and aligns with current goals of enhancing explainability in medical AI^18,19^.

Several Networks have already been trained that show good results in the detection of shunt valves^8–11^. Giancardo et al., using 416 X-ray images with the most common shunt valves, trained four different pipelines to identify the type of shunt valves, achieving an accuracy of up to 96%^8^. However, a radiologist needed to define an image window around the shunt valve beforehand. In contrast, our model offers the advantage of automatic object recognition, which independently identifies and assigns the shunt valve. In addition, our model demonstrated excellent performance, achieving a weighted average precision of 98.2%.

Other networks have also been successfully trained to identify CSF shunt valves. In their study, Sujit et al. developed a deep learning model that can identify implanted CSF shunt valves using photos taken with cell phone cameras from the screens of radiologists’ workstations^9^. This approach suggests a practical solution for the identification of shunt valves right at the radiologist’s workstation, achieving a very good mean accuracy of 95% and an average F1-Score of 0.95^9^. However, Sujit et al.‘s study was limited to differentiating between three shunt valve types and required manual preprocessing steps, such as defining the ROI. In contrast, our model enables a fully automated workflow and distinguishes between six different shunt valve types - Codman Certas, Codman Hakim, Codman Uni-Shunt, paediGAV, proGAV, and proGAV with gravitational unit - which represent the most frequently implanted valves in the underlying clinical cohort.

Compared to Rhomberg et al., who developed a highly accurate classification system based on CNNs^10^, our approach adds a clinically relevant layer of interpretability by integrating a CBRIA system. This mechanism automatically links each detected valve to a corresponding manufacturer reference image, enabling radiologists to visually verify the classification and directly determine the pressure setting. To our knowledge, this is the first study to combine deep learning-based object detection with such a reference image-based control mechanism, tailored specifically to clinical requirements. This integration streamlines the diagnostic workflow. Our method eliminates intermediary steps, such as capturing photos through third-party applications, and has the potential to be seamlessly integrated into the clinical imaging workflow as an automated recognition system in the future. This not only simplifies the identification of shunt valves but also offers the additional benefit of directly displaying the manufacturer’s image, enabling an easy comparison with the detected valve and a straightforward determination of the pressure setting. This approach is designed to reduce the time radiologists spend identifying shunt valve types and reading pressure levels. Moreover, our model performs both object detection and classification, automating the entire process, including the identification of ROI. The integration of manufacturer images in our method provides a unique control and safety mechanism. By automatically linking the detected valves with the corresponding manufacturer images, radiologists can immediately identify and correct potential misclassifications. This difference highlights the practical value of our method for clinical practice, as the combination of automatic detection and a safety mechanism not only simplifies the identification of valve types but also ensures the reliability of the results.

The evaluation of our object detection algorithm’s performance, measured by the weighted mAP values at an IoU threshold of 50%, resulted in an mAP value of 0.884. This value was not reported to demonstrate methodological innovation, but to indicate that the model’s predictions are indeed based on the correct anatomical target - the shunt valve. The location of the bounding box enables transparent verification of this focus. In the context of this application, visual traceability of the model’s decision is more relevant than precise localization as assessed by higher IoU thresholds.

While higher IoU thresholds such as AP_75_ and AP_90_ emphasize the accuracy of bounding box placement, they are less relevant to our primary goal - correct classification of valve type and robust reference image assignment. The clinical value lies in interpretable identification, not in submillimeter localization. Nonetheless, for completeness, we have included the AP_75_ and AP_90_ values in Supplementary Table S1. As expected, these values decrease with increasing thresholds. They were included to provide a more complete technical characterization of model performance.

Our study on the automatic detection of CSF shunt valves has some limitations. A major constraint is the limited availability and diversity of the training data, particularly for rare valve types, which impacts the model’s accuracy and robustness. To enhance robustness, we applied data augmentation techniques such as mosaic augmentation, random erasing, and geometric transformations. While no ablation studies were conducted, the high classification performance - especially for common valve types - indicates that augmentation likely improved generalizability. While this study investigated only a small group of shunt valves, it focused on the most implanted valves in our clinical setting. To overcome this, future studies should adopt a multicentric design to substantially increase the training dataset, particularly the variety of shunt valve types. Such an approach would improve the model’s generalizability and ensure broader applicability in clinical practice.

## Conclusion

The automated detection and classification of CSF shunt valves from X-ray images provides a framework that combines object detection with a class-based reference image assignment (CBRIA) system, offering a robust solution for identifying shunt valve types and linking them to manufacturer images. Our findings demonstrate high efficiency and precision in classifying CSF shunt valves. The integration of a safety mechanism allows for the immediate identification and correction of potential misclassifications, enhancing both reliability and accuracy. Furthermore, the matching with manufacturer images facilitates the straightforward determination of pressure levels, further streamlining the diagnostic workflow.

While our study highlights the potential of this framework, expanding the training dataset to include rare valve types and conducting multicentric studies could enhance its generalizability and broader clinical applicability.

## Supplementary Information

Below is the link to the electronic supplementary material.

Supplementary Material 1

## Data Availability

The datasets used and/or analyzed during the current single-center study are available from the corresponding author on reasonable request. To promote transparency, reproducibility, and further research, the full source code of the algorithm is publicly available at: https://github.com/JannisStraus/VPShuntDetector..

## Abbreviations

CBRIA: Class-based Reference Image Assignment
CNN: Convolutional neural network
COCO: Common Objects in Context
CSF: Cerebrospinal fluid
IoU: Intersection over union
mAP: Mean average precision
ROI: Region of interest
YOLOv8x: You Only Look Once, Version 8, Extra Large
