# Supplementary material for: Fully automated detection and identification of CSF shunt valves using YOLOv8 and a class-based reference image assignment as a safety mechanism
Source: Sci Rep. 2025 Nov 21;15:41188. doi: 10.1038/s41598-025-29201-0 (PMC12639084; doi:10.1038/s41598-025-29201-0)
Supplement: Supplementary file 1 — Supplementary Material 1 [file 41598_2025_29201_MOESM1_ESM.docx]

**Supplementary**

**Supplementary Table 1:** Detection performance of the object detection model by valve type, measured as Average Precision (AP) at IoU thresholds of 50%, 75%, and 90%, and mean AP across thresholds from 50% to 95% (mAP50–95).

|  | **n** | **AP_50_** | **AP_75_** | **AP_90_** | **mAP_50-95_** |
| --- | --- | --- | --- | --- | --- |
| Codman Certas | 138 | 0,919 | 0,161 | 0,004 | 0,363 |
| Codman Hakim | 133 | 0,949 | 0,078 | 0,001 | 0,333 |
| Codman Uni-Shunt | 8 | 0,253 | 0,093 | 0 | 0,116 |
| paediGAV | 2 | 0,268 | 0,160 | 0 | 0,152 |
| proGAV | 11 | 0,483 | 0,323 | 0,025 | 0,299 |
| proGAV gravitational unit | 3 | 0,028 | 0 | 0 | 0,004 |
| weighted mAP |  | 0,884 | 0,126 | 0,003 | 0,335 |

This table provides a detailed breakdown of detection performance per valve class. The AP metrics reflect the model’s ability to both localize and correctly classify CSF shunt valves. AP50 emphasizes correct detection with moderate localization accuracy, while AP75 and AP90 represent stricter localization criteria. The mAP50–95 corresponds to the mean AP over ten IoU thresholds from 0.50 to 0.95 (in 0.05 steps), following the COCO evaluation protocol. The final row shows the weighted average across all classes, using the number of validation samples per valve type as weights.
